# Gut microbiota-derived indole metabolites in colorectal cancer: potential links to PI3K/AKT signaling pathway

**DOI:** 10.3389/fmicb.2026.1913453

**Published:** 2026-09-21

**Authors:** Abrory A. C. Pramana, Alison Ho, Guanying B. Xu, Yuan-Xiang Pan, Hong Chen

**Affiliations:** 1 Division of Nutritional Sciences, College of Agricultural, Consumer, and Environmental Sciences, University of Illinois, Urbana, IL, United States; 2 Department of Biochemistry, Faculty of Medicine, Public Health, and Nursing, Universitas Gadjah Mada, Yogyakarta, Indonesia; 3 Department of Food Science and Human Nutrition, College of Agricultural, Consumer, and Environmental Sciences, University of Illinois, Urbana, IL, United States; 4 Cancer Center at Illinois, University of Illinois, Urbana, IL, United States

**Keywords:** CRC, gut, indoles, metabolites, PI3K/AKT

## Abstract

Gut inflammation associated with colorectal cancer (CRC) is closely linked to gut microbiota dysbiosis and altered microbial metabolites that disrupt intestinal homeostasis. Among these metabolites, indole derivatives produced through microbial tryptophan metabolism have emerged as important regulators of colon health. Recent evidence suggests that indole derivatives can influence gut barrier integrity, colon inflammation, and CRC progression. In this mini-review, we summarize current evidence regarding the role of indole derivatives in gut barrier regulation and CRC development. We also discuss their potential relationship with signaling pathways implicated in CRC progression, particularly the PI3K/AKT pathway, which may offer new perspectives on improving colon health and preventing inflammation-associated CRC.

## 1 Introduction

CRC is the third leading cause of mortality worldwide (Pramana et al., 2025; Wang et al., 2025). This cancer typically arises from a precancerous lesion, which, if not detected early, can further progress to invasive malignant cells (Tsai et al., 2025). Recently, the incidence of cancer among people under 50 years old, known as early onset of CRC (EO-CRC), has been increasing globally (Lopes-Junior, 2025). The factors, including genetic inheritance and environmental cues, have potentially contributed to driving this EO-CRC (Eraghieh Farahani et al., 2025; Lopes-Junior, 2025). Emerging evidence suggests that gut microbiota dysbiosis, altered gut metabolites, and chronic colon inflammation, such as in IBDs, play a strong role in driving CRC progression (Cui, 2025; Eraghieh Farahani et al., 2025; Pramana et al., 2025; Tsai et al., 2025).

The gut microbiota is a complex community of microorganisms, mainly bacteria, viruses, and fungi, residing in the gastrointestinal tract and maintaining the host’s intestinal homeostasis through symbiosis (Cui, 2025; Yang et al., 2025). In this complex ecosystem, gut bacteria are dominant, and the main phyla colonizing the gastrointestinal tract include Firmicutes, Bacteroidetes, Actinobacteria, and Proteobacteria (Yang et al., 2025). Gut dysbiosis is a condition of altered gut microbiota composition and function (Cui, 2025). Gut dysbiosis has been associated with various health issues, affecting both the gut and the brain (Seneviwickrama et al., 2025). Recent studies have reported gut microbiota dysbiosis, including depletion of beneficial bacteria such as *Bifidobacterium* sp., *Lactobacillus* sp., and *Dubosiella* sp., in animal models of CRC (Liu et al., 2025; Pramana et al., 2025). A similar pattern is also observed in patients with colorectal cancer (Seneviwickrama et al., 2025; Tudorache et al., 2025). Metabolomic analysis has revealed that gut microbial metabolites, i.e., indole derivatives, may mediate crosstalk between the microbiota and the host during CRC progression (Qu et al., 2023; Cao et al., 2025; Griffith et al., 2025).

The PI3K/AKT signaling pathway is one of the pathways commonly dysregulated in CRC patients (Papadatos-Pastos et al., 2015). This pathway acts as the crucial signaling cascade involved in cell proliferation, apoptosis, migration, invasion, tumor angiogenesis, and anti-cancer resistance treatment (Chen et al., 2026). The activation of the AKT/protein kinase B (PKB) is mainly the inducer of cell cycle and proliferation through the downstream phosphorylation of specific regulatory proteins, such as cyclin-dependent kinases (CDKs), leading to the unchecking of cell proliferation (Leiphrakpam et al., 2025). Therefore, targeting the PI3K/AKT signaling pathway could provide a novel approach to mitigate uncontrolled cell cycle proliferation during CRC progression (Ahmad et al., 2013).

In this mini-review, we presented recent evidence on the role of indole-derivative metabolites in enhancing gut barrier function and their impact on CRC progression. Notably, this review highlights the potential of indole derivatives to modulate CRC via the PI3K/AKT signaling pathway and identifies a novel therapeutic target to prevent CRC progression.

## 2 Indole derivatives: gut-microbial tryptophan metabolism

Tryptophan is an essential amino acid supplied from the diet and is utilized for protein synthesis (Gao et al., 2020). The recommended dose of tryptophan is 4 mg/kg/day, and no adverse effects have been reported with excessive intake (Agus et al., 2018; Gupta et al., 2023). Tryptophan is absorbed in the small intestine and circulates in the blood bound to albumin (Gupta et al., 2023). Tryptophan in the gastrointestinal tract can be metabolized by the host and gut microbiota, affecting host metabolism and homeostasis (Sinha et al., 2024). In the host, tryptophan is metabolized through two pathways, including (1) the kynurenine pathway, in the epithelial and immune cells via indoleamine 2,3-dioxygenase (IDO) 1, and (2) the serotonin pathway, in the enterochromaffin cells via tryptophan hydroxylase (TPH) 1 (Agus et al., 2018; Gao et al., 2020). Meanwhile, gut microbiota in the gastrointestinal tract metabolizes tryptophan into indole and its derivatives, including indolelactic acid (ILA), indolepropionic acid (IPA), indolealdehyde (IAld), indoleacrylic acid (IAcrA) (Gupta et al., 2023), and indole-3-carboxaldehyde (ICA) (Lu et al., 2023).

The gut microbiota expresses enzymes that help metabolize tryptophan (Roager and Licht, 2018). Importantly, gut microbiota contribute to tryptophan metabolism into indole and its derivatives, such as indican, skatole, and tryptamine (Dehhaghi et al., 2019). For instance, *Clostridium sporogenes* contains phenyllactate dehydratase, encoded by the *fldC* gene, that converts tryptamine, a tryptophan-derived product, into IPA (Dodd et al., 2017). *Peptostreptococcus*, another genus of the gut microbiota, harbors a unique phenyllactate dehydratase gene cluster (*fldAIBC*) that also metabolizes tryptophan and other aromatic amino acids into IPA, IAcrA, and 3-hydroxyphenyl propionic acid (Wlodarska et al., 2017). A study in mice colonized with *Parasutterella* increased the abundance of indole-derived metabolites from tryptophan metabolism, including indole-2-carboxylic acid, 3-methyldioxyindole, and indole-3-carboxylic acid (Ju et al., 2019), suggesting the potential role of gut microbiota colonization in shaping the host’s gut metabolite profile. Interestingly, recent evidence indicates that indole derivatives produced by the gut microbiota affect the host’s gastrointestinal physiology, including maintenance of epithelial tight junctions, modulation of the immune response, protection against pathogens, and regulation of inflammation and metabolic disorders (Sinha et al., 2024). Those findings further support the role of the gut microbiota in metabolizing aromatic amino acids, particularly tryptophan, into derivative metabolites that support gut homeostasis.

## 3 The role of indole derivatives in enhancing gut barrier integrity

Evidence suggests that damage to barrier integrity, especially during colon inflammation, breaks down the gut barrier and enhances CRC progression (Genua et al., 2021). The gut barrier, composed of tight junction proteins (TJPs), maintains epithelial integrity in the mucosal layer of the gut and prevents harmful substances, such as pathogenic bacteria and their toxins, from crossing it (Liu et al., 2023). Growing evidence shows that indole-derived metabolites can enhance gut barrier integrity. The IPA study showed increased gut barrier integrity in Caco2/HT29 co-culture after lipopolysaccharide (LPS) induction (Li et al., 2021). They found that IPA restored gut barrier integrity by upregulating tight junction proteins (TJPs), including Claudin-1, Occludin, and ZO-1. Additionally, IPA treatment increased mucus production, further restoring the physical barrier. Another study reported that increased concentrations of indole-3-acetic acid (IAA) and IAld in a mouse model of colitis were associated with enhanced gut barrier integrity (Yang et al., 2021). In their study, supplementation with turmeric polysaccharides increased IAA and IAld in the mouse cecum, increased aryl hydrocarbon receptor (AhR) protein levels, and restored gut barrier integrity by upregulating ZO-1 and Occludin in the colon. Consistent with these findings, supplementation with indole-3-carboxaldehyde (ICA) attenuated pouchitis, an ileal inflammatory condition during colitis, in a recent study (Zhang et al., 2025). ICA significantly increased IL-22 and decreased TNF-α gene expression, induced pSTAT3 activation, and decreased lymphocyte counts. Collectively, these data support the potential benefit of gut metabolites derived from bacterial tryptophan metabolism in enhancing gut barrier integrity and potentially decreasing CRC progression.

## 4 The role of indole derivatives from gut microbial metabolites in CRC progression

Currently, indole derivatives produced by gut microbial metabolism of tryptophan have been shown to influence CRC progression (Jia et al., 2024). Several indole derivatives show tumor-suppressive activity. For example, ILA produced by *Lactobacillus gallinarum*, has been shown to protect against colorectal tumorigenesis (Sugimura et al., 2021). They identified increased ILA in stool samples from *Apc*^Min/+^ mice treated with *L. gallinarum*, corresponding to an increased abundance of this indole derivative in *L. gallinarum*-free cultured supernatant. Mechanistically, they found that ILA induces apoptosis *in vitro* and that disruption of AhR signaling (with an AhR antagonist) abolishes ILA’s suppression of CRC. Notably, another study reported that ILA also inhibits CRC cells via the p-STAT3-HK2 axis in an AhR-independent manner, suggesting a distinct downstream mechanism by which ILA suppresses CRC progression (Zhou et al., 2025). In addition, indoxyl sulfate (IS), another indole derivative, exhibited potent activity against CRC cell lines (Dalal et al., 2023). In their study, IS treatment induced cells to halt mitosis, increasing the number of cells in the G2/M phase of the cell cycle compared with controls. Additionally, they observed a higher percentage of apoptotic cells in IS-treated cells than in controls. Similarly, indole-3-acetic acid (IAA) exerted anti-proliferative effects on the Caco-2 CRC cell line (Tomii et al., 2023). They found that Tlr4 induction, but not AhR, contributed to the activation of c-Jun N-terminal kinase (JNK), leading to an anti-proliferative effect. In addition, ICA produced by *Dubosiella newyorkensis* has been reported to reduce tumor numbers compared with CRC-group animals (Yang et al., 2026). These findings support the role of indole derivatives in suppressing CRC progression.

Furthermore, these data support a potential link between indole derivatives and the regulation of proliferation and the cell cycle, particularly through the PI3K/AKT signaling pathway.

## 5 The role of indole derivatives from gut microbial metabolites in the PI3K/AKT signaling pathway

The PI3K/AKT signaling pathway is one of the most dysregulated pathways in cancer and has been investigated as a therapeutic target to suppress cancer progression (Popolo et al., 2017; Leiphrakpam et al., 2025). This pathway is also crucial in cell cycle regulation (Chen et al., 2026). Although direct evidence for indole derivatives suppressing CRC progression is limited, emerging studies in gastrointestinal models suggest these metabolites may modulate the PI3K/AKT signaling pathway, including p21 and PTEN. These studies also offer mechanistic hypotheses warranting further investigation in CRC. A study of indole-3-carboxylic acid found enhanced cellular senescence in a CRC cell line treated with doxorubicin (Zhou et al., 2024). The authors discovered that supplementation with indole-3-carboxylic acid inhibits cell proliferation and promotes cell-cycle arrest by increasing p21 protein levels. The p21 is a tumor suppressor that induces cell cycle arrest (Ooi et al., 2024), and its upregulation has been associated with decreased phosphorylation of AKT (pAKT) (Chang et al., 2019).

Supernatant from *L. plantarum*, an isolated probiotic, exhibited apoptotic activity against the gastric cancer cell line (Maleki-Kakelar et al., 2020). Their study found that media-free *L. plantarum* increases gene expression of *BAX*, *PTEN*, and *TLR4* and decreases *AKT* expression. The apoptotic activity of AGS cells, a gastric cancer cell line, was significantly increased after treatment with media-free *L. plantarum;* however, the authors did not identify specific *L. plantarum* metabolites that induce apoptosis and reduce AKT activity. 2-Methyl-7-phenylindole is dominantly produced in the culture medium of *Limosilactobacillus fermentum* and exhibits anticancer activity against the HT-29 CRC cell line via the PTEN/PI3K/AKT pathway (Ahrabi et al., 2025). They demonstrated that this metabolite induces apoptosis in the CRC cell line. Furthermore, gene expression analysis revealed downregulation of *AKT*, followed by upregulation of *CASP3* and *PTEN*. Molecular docking analysis identified a strong binding affinity between 2-Methyl-7-phenylindole and AKT, suggesting that this metabolite may inhibit proliferation in CRC cell lines via the AKT signaling pathway. Additionally, IPA has been reported to downregulate proinflammatory cytokines in co-cultures of HT-29 and Caco-2 cells in response to lipopolysaccharide (LPS) treatment (Li et al., 2021). They found that IPA treatment in the induced inflammation in the CRC cell line downregulated the gene expression of *PI3K*, *AKT*, and *mTOR*. Indole-3-carbaldehyde (ICAld) from *L. reuteri* has been reported to suppress diffuse large B-cell lymphoma (Zhang et al., 2026). ICAld has been reported to decrease the protein levels of phospho-PI3K (pPI3K), phospho-AKT (pAKT), and phospho-mTOR (pmTOR), and to increase the protein levels of apoptosis-related proteins such as BAX and cleaved CASPASE 3.

Overall, the current evidence suggests a potential regulatory relationship rather than a direct mechanism, highlighting the need for future studies to validate the molecular mechanism in CRC models, both *in vivo* and *in vitro*.

## 6 Discussion

Indoles and their derivatives produced by gut microbiota metabolism are unique metabolites whose functions have attracted growing interest in the context of colon health, particularly during CRC progression. However, research in this area remains limited. The indole ring serves as the core structure of several important compounds, such as serotonin, tryptophan, and melatonin, that regulate cell signaling and neurotransmission (Kulyal et al., 2025). In the colon, where the gut microbiota reach their highest abundance in the human body, indole derivatives also play an important role in microbial signaling, including spore formation, plasmid stability, drug resistance, and toxicity (Ye et al., 2022). Thus, indole derivatives may affect the host and the composition and function of gut microbiota.

In this review, we discuss current evidence on the effects of indole derivative metabolites from the gut microbiota on gut health, particularly colon health, as depicted in Figure 1 and summarized in Table 1. These metabolites may play an important role in maintaining colon health by enhancing mucus production and reducing gut permeability, thereby lowering CRC risk. Additionally, these metabolites also modulate the cell cycle, potentially affecting the PI3K/AKT signaling pathway and inhibiting CRC cell proliferation.

**Figure 1:**
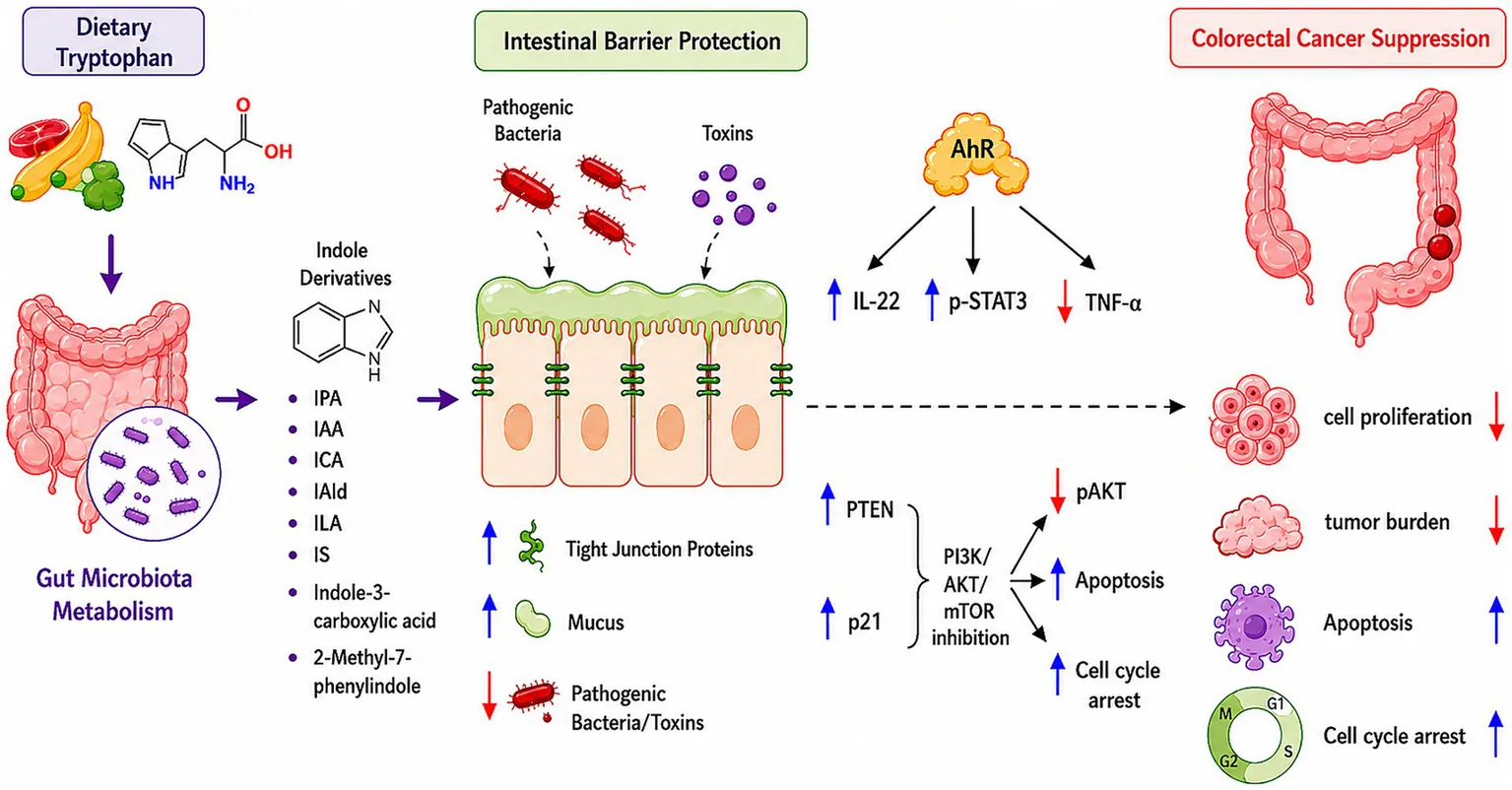
The role of indole derivatives in colon homeostasis. Supplementation of indole derivatives promotes mucus production and enhances gut barrier integrity. In addition, indole derivatives also affect CRC. Indole derivatives regulate pAKT, a key regulator in the PI3K/AKT signaling pathway in CRC progression, which decreases cancer proliferation. Created in BioRender. Pramana, A. A. C. (2026), https://BioRender.com/u2likgd.

**Table 1:** The effects of indole derivative gut metabolites on the gut barrier and CRC progression.

Metabolite name	Target cell type or tissue	Major receptor or signal pathway	Biological function	Effect on CRC progression	Experimental model	References
(a) Indole derivative metabolites and gut barrier integrity						
Indole-propionic acid (IPA)	LPS-treated Caco-2 and HT29 colonic epithelial cells	Tight junction proteins	↑MUC4, ↑MUC2, ↑TFF3, ↑RELMβ in HT29; ↑Claudin-1, ↑Occludin, ↑ZO-1 in Caco2	↑Gut barrier integrity; ↓CRC risk	*In vitro*	Li et al. (2021)
Indole-3-aldehyde (Iald)	Mouse colon from DSS-induced colitis model	AhR	↑AhR protein levels; ↑Claudin-1, Occludin, and ZO-1 expression; restored epithelial tight junctions and gut barrier integrity	↑Gut barrier integrity; ↓CRC risk	*In vivo*	Yang et al. (2021)
Indole-3-acetic acid (IAA)	Mouse colon from DSS-induced colitis model	AhR	↑AhR protein levels; ↑Claudin-1, Occludin, and ZO-1 expression; restored epithelial tight junctions and gut barrier integrity	↑Gut barrier integrity; ↓CRC risk	*In vivo*	Yang et al. (2021)
Indole-3-carboxaldehyde (ICA)	Ileum from DSS-induced colitis mouse model	IL-22; STAT3	↑IL-22; ↓TNF-α gene expression; ↑pSTAT3 activation; ↓lymphocyte counts; attenuated colitis and restored intestinal barrier integrity	↑Gut barrier integrity; ↓Inflammation associated with CRC	*In vivo*	Zhang et al. (2025)
(b) Indole derivative metabolites and colorectal cancer progression						
Indole-3-lactic acid (ILA)	Colon tissue of *Apc*Min/+ mice and HCT116, LoVo and NCM460 colonic epithelial cells	AhR	↑Apoptosis	↓Tumor Formation	*In vivo*; *in vitro*	Sugimura et al. (2021)
Indole-3-lactic acid (ILA)	Colon tissue of mice and the MC38 and HCT116 CRC cell lines	AhR (independent)-p-STAT3-HK2	↓Tumor size and volume in an animal model; ↑Apoptotic cells, ↓HK2, ↓p-STAT3 in the CRC cell lines	↓CRC Progression	*In vitro*	Zhou et al. (2025)
Indoxyl sulfate	HT-29 and HCT116 CRC cell lines and Balb/c mouse model	n.a.	↓Cell viability, ↓Membrane integrity, ↑Apoptosis, ↓Colony formation, ↑Cell number in the G2/M phase in CRC cell lines; No induction of inflammation in normal colonic cells in a mouse study	↓CRC Progression	*In vivo*; *in vitro*	Dalal et al. (2023)
Indole-3-acetic acid (IAA)	Caco-2 CRC cells	Tlr4-JNK	↑phospho-ERK and phospho-JNK	↓CRC Progression	*In vitro*	Tomii et al. (2023)
Indole-3-carboxaldehyde (ICA)	C57BL/6 J and *eIF6* heterozygous (eIF6+/−) mice model	n.a.	↓Tumor numbers	↓CRC Progression	*In vivo*	Yang et al. (2026)
(c) Indole derivative metabolites and PI3K/AKT signaling						
Indole-3-carboxylic acid	LS180 CRC cell line and rat model (xenograft)	PI3K/AKT	↑Senescence-associated heterochromatin foci (SAHF), ↑p21 protein levels both in the CRC cell line and xenograft, and ↓reduced the proportion of G1/S phase cells and ↑percentage of G2 phase cells.	↓CRC progression	*In vitro*	Zhou et al. (2024)
Indole-3-carbaldehyde (ICAld)	SU-DHL-4 and OCI-LY3 (diffuse large B-cell lymphoma cells line)	AhR and PI3K/AKT/mTOR	↑AhR, ↑BAX, ↑cleaved-CASPASE3, ↓BCL-2, ↓p53; ↓phospho-PI3K, ↓phospho-AKT, ↓phospho-mTOR	↓Tumor progression	*In vitro*; *in vivo*	Zhang et al. (2026)
2-Methyl-7-phenylindole	HT-29 CRC cell line	PTEN/PI3K/AKT	↑*PTEN* and *CASPASE3*; ↓*AKT* gene expression ↑Apoptosis	↓CRC Progression	*In vitro*	Ahrabi et al. (2025)
Indole-propionic acid (IPA)	LPS-treated Caco-2 and HT29 colonic epithelial cells	PI3K/AKT/mTOR	↓*TNF-α*, *IL-8*, *IL-6*, *PI3K*, *AKT*, *mTOR* in cells treated with LPS treatment	↓CRC risk	*In vitro*	Li et al. (2021)

Indole derivative metabolites are the ligands that activate AhR protein (Agus et al., 2018). Activation of this protein regulates cellular processes, including the cell cycle, apoptosis, immunomodulation, and barrier function (Yin et al., 2016; Li et al., 2025). A study in a colitis mouse model showed that an AhR antagonist abolished the protective effects of tryptophan supplementation by suppressing antimicrobial peptide production, decreasing goblet cells, and inducing pro-inflammatory cytokines such as IL-6, IL-1β, and TNF-α (Li et al., 2026). Aligning with this data, high-fat diet consumption in an intestinal-epithelial-specific (IEC) AhR knockout (KO) mouse has been found to promote crypt cell proliferation and enhance CRC progression (Garcia-Villatoro et al., 2020). These data suggest that AhR mediates colon homeostasis during inflammation and CRC progression; therefore, targeting indole derivative metabolites as AhR ligands may offer a new opportunity to prevent CRC progression.

Clinical trials of indole derivative supplementation are limited, with most research conducted in animal models and *in vitro* using established cell lines. Although ILA, an indole derivative, has been reported to be significantly reduced in CRC patients, limitations remain, including the safety and effectiveness of ILA supplementation in humans (Zhou et al., 2025). These limitations further highlight the need for future investigation on how indole derivatives may affect CRC signaling pathways, such as the PI3K/AKT pathway. Furthermore, this could be an opportunity to explore the use of prebiotics or postbiotics to modulate the reduced abundance of indole derivatives during gut inflammation associated with CRC, potentially elucidating the interconnection among gut health, indole derivatives, and their impact on the host to decrease CRC risk.

In conclusion, current evidence supports the emerging concept that microbiota-derived indole metabolites may serve as critical therapeutic targets for improving colon health and potentially reducing CRC risk.
